# Diabetes risks and outcomes in chronic obstructive pulmonary disease patients: Two nationwide population-based retrospective cohort studies

**DOI:** 10.1371/journal.pone.0181815

**Published:** 2017-08-16

**Authors:** Chao-Shun Lin, Chih-Chung Liu, Chun-Chieh Yeh, Yi-Cheng Chang, Chi-Li Chung, Hsin-Long Lane, Chun-Chuan Shih, Ta-Liang Chen, Chien-Chang Liao

**Affiliations:** 1 Department of Anesthesiology, School of Medicine, College of Medicine, Taipei Medical University Hospital, Taipei, Taiwan; 2 Anesthesiology and Health Policy Research Center, Taipei Medical University Hospital, Taipei, Taiwan; 3 Department of Anesthesiology, Taipei Medical University, Taipei, Taiwan; 4 Department of Surgery, China Medical University Hospital, Taichung, Taiwan; 5 Department of Surgery, University of Illinois, Chicago, United States of America; 6 Division of Endocrinology, Department of Medicine, National Taiwan University Hospital, Taipei, Taiwan; 7 Division of Pulmonary Medicine, Department of Internal Medicine, Taipei Medical University Hospital, Taipei, Taiwan; 8 School of Chinese Medicine for Post-Baccalaureate, College of Medicine, I-Shou University, Kaohsiung, Taiwan; 9 School of Chinese Medicine, College of Chinese Medicine, China Medical University, Taichung, Taiwan; 10 Department of Anesthesiology, Shuan Ho Hospital, Taipei Medical University, New Taipei City, Taiwan; National Yang-Ming University, TAIWAN

## Abstract

**Objective:**

The relationship between chronic obstructive pulmonary disease (COPD) and diabetes remains incompletely understood. This study evaluated diabetes risk and post-diabetes outcomes in COPD patients with and without exacerbations.

**Methods:**

We identified 4671 adults newly diagnosed with COPD exacerbations and 9342 adults newly diagnosed with COPD without exacerbations during 2000–2008 using Taiwan’s National Health Insurance Research Database. A comparison cohort of 18684 adults without COPD, matched by age and sex, was randomly selected from the same dataset for the control group. Diabetes events during 2000–2013 were ascertained from medical claims during the follow-up period. Adjusted hazard ratios (HRs) and 95% confidence intervals (CIs) of diabetes associated with COPD with or without exacerbations were calculated. We conducted another nested cohort study of 395516 patients with diabetes hospitalization during 2002–2013 and calculated adjusted odds ratios (ORs) and 95% CIs of histories of COPD and COPD exacerbations associated with adverse events after diabetes admission.

**Results:**

During the follow-up period, the incidences of diabetes for patients without COPD and for patients with COPD without or with exacerbations were 3.4, 4.1 and 7.4 per 1000 person-years, respectively (P < 0.0001). Increased risk of diabetes for patients with COPD without exacerbations (HR 1.09, 95% CI 1.02–1.17) and COPD with exacerbations (HR 2.18, 95% CI 1.88–2.52) was noted. Post-diabetes pneumonia (OR 3.28, 95% CI 3.13–3.43), intensive care admission (OR 1.32, 95% CI 1.26–1.39) and mortality (OR 2.06, 95% CI 1.88–2.25) were associated with COPD exacerbations.

**Conclusion:**

Prevention and intervention strategies for diabetes and post-diabetes outcomes are needed for this susceptible population.

## Introduction

In the United States, diabetes is one of the leading causes of adult death and disability. It also represents a large and rapidly growing economic burden, with an estimated cost of US$245 billion in 2012 [1,2]. Although diabetes’ epidemiology, pathogenesis, treatment guidelines and prevention programs have been well established over 204 years [3], it remains a pandemic disease that will reach an estimated global prevalence of 4.4% by 2030 [4].

Type 2 diabetes is considered a common comorbidity for patients with chronic obstructive pulmonary disease (COPD) and reduced lung function [5–10]. Epidemiological investigations show that diabetes is much more likely in patients with COPD than in control subjects [11,12]. A controversial population-based study using an Italian database found patients with COPD had an increased risk for diabetes compared to non-COPD subjects [13,14].

Although the association between COPD and diabetes risk was reported in previous studies [11–16], the risk of diabetes for COPD patients in previous studies were limited by cross-sectional study design [11–14], lack of adequate control subjects [11–14], poor adjustment for potential confounders [15,16], short follow-up and incorrect selection of COPD cases [15]. The impact of COPD exacerbations on diabetic patients’ risk and outcomes is also unclear.

Based on the context described above, we hypothesized that patients with COPD may have increased the risk and adverse outcomes of diabetes. Using claims data from Taiwan’s National Health Research Database [6,7], we conducted a nationwide longitudinal cohort study to assess the risks of diabetes, post-diabetes mortality and complications in patients with COPD.

## Methods

### Source of data

Reimbursement claims used in this study were collected from the National Health Insurance Research Database. This insurance program was implemented in March 1995 and covers more than 99% of Taiwan’s 23 million residents. The National Health Research Institutes established this database to record beneficiaries’ medical services, including inpatient and outpatient demographic characteristics, physicians’ primary and secondary diagnoses, treatment procedures, prescriptions and medical expenditures. Research articles based on this database have been accepted in prominent scientific journals worldwide [6,7]. To protect personal privacy, the database was decoded and patient identifications were scrambled for further public access for this research. This study was evaluated and approved by the Joint Institutional Review Board of Taipei Medical University (TMU-JIRB-201605049) and E-DA Hospital (EDA-JIRB-2017004).

### Study design and population

We used the National Health Insurance Research Database to perform two nationwide, population-based retrospective cohort studies. Using the database’s representative sample of one million beneficiaries, we conducted a retrospective cohort study of 9,342 COPD patients without exacerbations and 4671 patients with newly diagnosed COPD exacerbations with frequency matching by age and sex (COPD: COPDe = 2:1). We defined COPD patients as follows: people had at least two medical visits for outpatient care with physician’s primary diagnosis of COPD within one year. We defined patients with COPD exacerbations as follows: people received physician’s care due to COPD in the hospitalization ward or emergency room These definitions of COPD and COPD exacerbations were based on previous reports [17–19,20,21]. For comparison, 18,684 frequency-matched individuals without COPD were selected (controls: COPDe = 4:1). These three cohorts, with subjects aged ≥40 years, were established between January 1, 2000, and December 31, 2005, and then followed until December 31, 2013. We calculated person-years during the follow-up period for each participant until diagnosis of diabetes or until censored because of death, withdrawal from the insurance system, or loss to follow-up. The non-COPD group included the remaining people who did not develop COPD during the follow-up period.

Using a diabetes cohort consisting of all incident diabetes patients among the total population of 23 million people from the National Health Insurance Research Database, we identified 395,516 new-onset diabetes patients hospitalized during 2000–2013. We compared sociodemographics, co-morbidities and medications for diabetes patients with no COPD, COPD, and COPDe. Risks of pneumonia, intensive care and mortality during diabetes admission were also estimated.

### Measures and definition

We identified income status and defined low-income patients as those qualifying for waived medical copayment, which was verified by the Bureau of National Health Insurance. *The International Classification of Diseases*, *Ninth Revision*, *Clinical Modification* (ICD-9-CM) was used to define chronic obstructive pulmonary disease (ICD-9-CM 491, 492, 496) [17,18], diabetes (ICD-9-CM 250), co-morbidities and post-diabetes complications. Co-morbidities included hypertension (ICD-9-CM 401–405), mental disorders (ICD-9-CM 290–319), ischemic heart disease (ICD-9-CM 410–414), stroke (ICD-9-CM 430–438), liver cirrhosis (ICD-9-CM 571), hyperlipidemia (ICD-9-CM 272.0, 272.1, and 272.2), heart failure (ICD-9-CM 428), anemia (ICD-9-CM 280–285), Parkinson’s disease (ICD-9-CM 332), atrial fibrillation (ICD-9-CM 427.31), and peripheral vascular disease (ICD-9-CM 443). Renal dialysis was identified by administration code (D8, D9). In-hospital 30-day mortality after the index diabetes admission was considered the primary outcome, and post-diabetes pneumonia (ICD-9-CM 480–486), intensive care, length of hospital stay and medical expenditure were considered secondary outcomes in the nested cohort study. We also considered the impact of invasive respiratory treatments on diabetes risk and outcomes, and these treatments included endotracheal tube insertion, tracheal stent insertion, tracheostomy, laryngotracheal reconstruction, repair of tracheobronchial tree, and endobronchial dilatation.

### Statistical analyses

Using the analysis of chi-square tests, we compared sociodemographic factors (such as age, sex and low income), co-morbidities (such as hypertension, mental disorders, liver cirrhosis, stroke, hyperlipidemia, heart failure, anemia, atrial fibrillation, peripheral vascular disease and renal dialysis), and medications (such as anticoagulant, anti-platelet agents and lipid-lowering agents) for people with no COPD, with COPD or with COPDe. The adjusted hazard ratios (HRs) and 95% confidence intervals (CIs) of diabetes associated with COPDe were calculated using multivariate Cox proportional hazard models. In the further stratified analysis, the adjusted HRs and 95% CIs of diabetes associated with COPD or COPDe were also calculated in both sexes and all age groups.

In the nested cohort study, analysis of chi-square tests compared differences in sociodemographics, co-morbidities and medications for diabetes patients in the three groups. By using multivariate logistic regressions, we calculated adjusted odds ratios (ORs) and 95% CIs for risks of pneumonia, intensive care and mortality after diabetes. The mean length of stay and medical expenditure were also compared by analysis of variance for diabetic patients without COPD or with COPD or COPDe.

## Results

After matching by age and sex among cohorts without COPD, with COPD and COPDe, proportionately more patients with COPDe had low-income status, hypertension, mental disorders, ischemic heart disease, stroke, liver cirrhosis, hyperlipidemia, heart failure, anemia, Parkinson’s disease, atrial fibrillation, peripheral vascular disease and renal dialysis, compared with people without COPD (p<0.0001). Use of medications such as anticoagulants, anti-platelet agents and lipid-lowering agents was also higher in patients with COPDe than in those without COPD (Table 1) (p<0.0001).

**Table 1:** Baseline characteristics of people with and without COPD.

	No COPD N = 18684		COPD N = 9342		COPDe N = 4671		*P* value
Sex	n	(%)	n	(%)	n	(%)	1.0000
Female	9256	(49.5)	4628	(49.5)	2314	(49.5)	
Male	9428	(50.5)	4714	(50.5)	2357	(50.5)	
Age, years							1.0000
40–49	5064	(27.1)	2532	(27.1)	1266	(27.1)	
50–59	5292	(28.3)	2646	(28.3)	1323	(28.3)	
60–69	5744	(30.7)	2872	(30.7)	1436	(30.7)	
70–79	2584	(13.8)	1292	(13.8)	646	(13.8)	
Low income	289	(1.6)	295	(3.2)	231	(5.0)	<0.0001
Co-morbidities							
Hypertension	8667	(46.4)	5405	(57.9)	2803	(60.0)	<0.0001
Mental disorder	5296	(28.4)	4225	(45.2)	2213	(47.4)	<0.0001
Ischemic heart disease.	3140	(16.8)	2900	(31.0)	1621	(34.7)	<0.0001
Stroke	2941	(15.7)	2095	(22.4)	1324	(28.4)	<0.0001
Liver cirrhosis	3316	(17.8)	2628	(28.1)	1151	(24.6)	<0.0001
Hyperlipidemia	3948	(21.1)	2841	(30.4)	1142	(24.5)	<0.0001
Heart failure	511	(2.7)	612	(6.6)	555	(11.9)	<0.0001
Anemia	1139	(6.1)	858	(9.2)	416	(8.9)	<0.0001
Parkinson's disease	355	(1.9)	245	(2.6)	166	(3.6)	<0.0001
Atrial fibrillation	209	(1.1)	236	(2.5)	161	(3.5)	<0.0001
Peripheral vascular disease	399	(2.1)	331	(3.5)	156	(3.3)	<0.0001
Renal dialysis	232	(1.2)	131	(1.4)	117	(2.5)	<0.0001
Anticoagulants	540	(2.9)	450	(4.8)	307	(6.6)	<0.0001
Anti-platelet agents	7884	(42.2)	5461	(58.5)	2965	(64.5)	<0.0001
Lipid-lowering agents	5660	(30.3)	3732	(40.0)	1877	(40.2)	<0.0001

COPD = chronic obstructive pulmonary disease; COPDe = chronic obstructive pulmonary disease with exacerbations.

**Fig 1:**
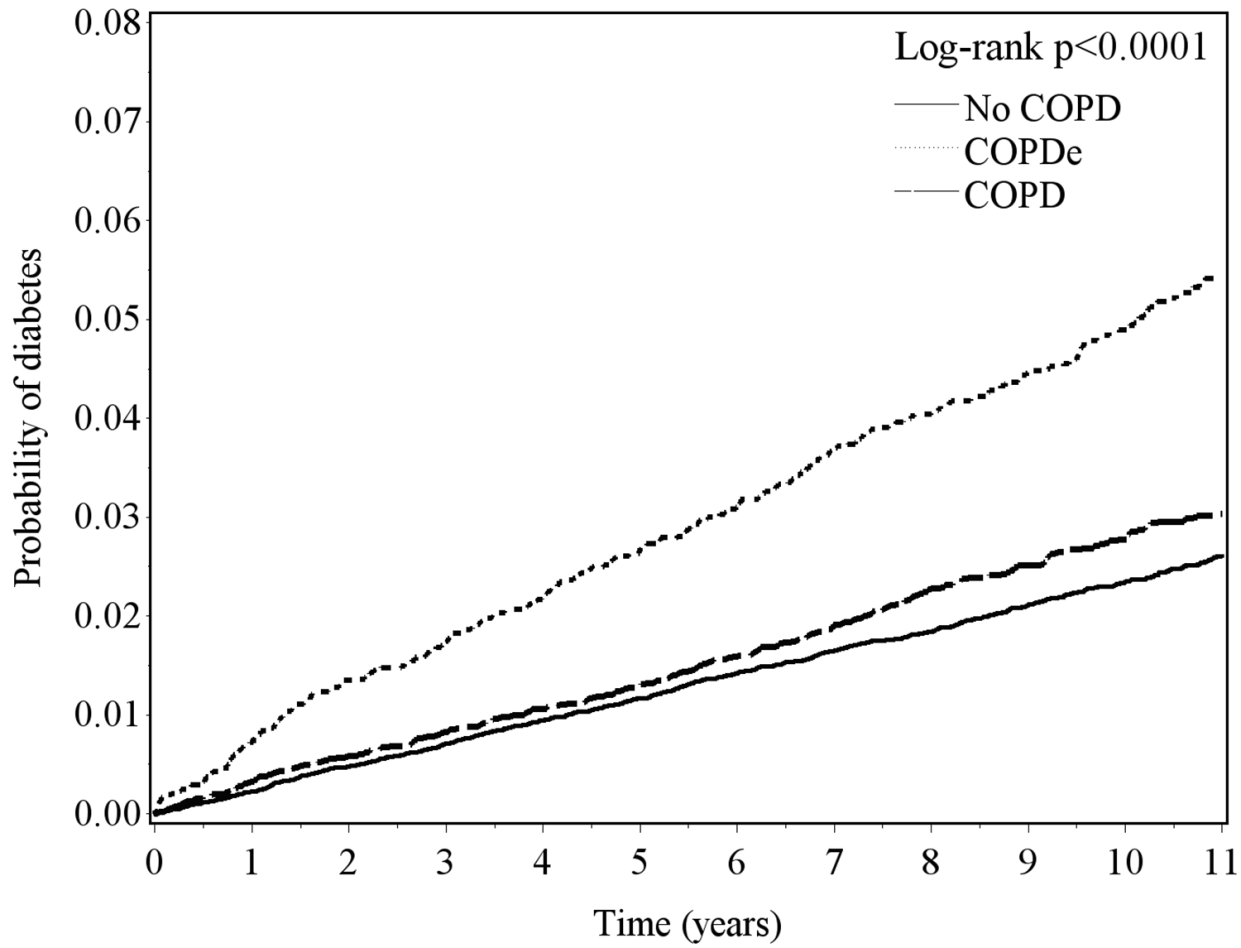
Probability of diabetes risk estimated using the Kaplan-Meier method for people with no COPD, COPD, and COPDe. Compared with non-COPD cohort or COPD cohort, patients with COPDe showed significantly increased probability of developing diabetes during the follow-up years (log-rank test, p<0.0001).

Table 2 shows a higher incidence of diabetes in patients with previous COPD and COPDe than those without COPD (4.1 and 7.4 vs. 3.4 per 1000 person-years, p<0.0001) during the follow-up period. The corresponding HRs for diabetes associated with COPD or COPDe were 1.09 (95% CI, 1.02–1.17) and 2.18 (95% CI, 1.88–2.52), respectively. The association between COPDe and diabetes risk was significant in females (HR, 2.11; 95% CI, 1.72–2.58), males (HR, 2.27; 95% CI, 1.83–2.82) and people in all age groups, specifically 40–49 years (HR, 3.73; 95% CI, 2.41–5.79), 50–59 years (HR, 2.88; 95% CI, 2.16–3.86), 60–69 years (HR, 1.71; 95% CI, 1.35–2.16) and 70–79 years (HR, 1.73; 95% CI, 1.23–2.42). HRs for diabetes risk associated with COPDe for people with 0, 1, 2, ≥3 co-morbidities were 2.68 (95% CI 1.74–4.11), 2.52 (95% CI 1.86–3.42), 2.19 (95% CI 1.65–2.90) and 1.81 (95% CI 1.45–2.26) respectively. Compared with the non-COPD cohort or the COPD cohort (Fig 1), patients with COPDe showed a significantly increased probability of developing diabetes during the follow-up years (log-rank test, p<0.0001). The diabetes risk associated with respiratory invasive treatment for patients with COPDe (HR 1.47, 95% CI 0.95–2.52) was not significant.

**Table 2:** Risk of diabetes for patients with and without COPD by sex and age.

		n	Events	Person-years	Incidence^b^	HR	(95% CI)^a^
No COPD		18684	563	167874	3.4	1.00	(reference)
COPD		9342	360	87209	4.1	1.09	(1.02–1.17)
COPDe		4671	308	41658	7.4	2.18	(1.88–2.52)
COPDe treatment^c^		646	23	3289	7.0	1.47	(0.95–2.26)
Female	No COPD	9256	302	85915	3.5	1.00	(reference)
	COPD	4628	188	43903	4.3	1.08	(0.99–1.19)
	COPDe	2314	162	21576	7.5	2.11	(1.72–2.58)
Male	No COPD	9428	261	81959	3.2	1.00	(reference)
	COPD	4714	172	43306	4.0	1.10	(0.99–1.22)
	COPDe	2357	146	20082	7.3	2.27	(1.83–2.82)
40–49 years	No COPD	5064	42	50281	0.8	1.00	(reference)
	COPD	2532	47	25346	1.9	1.37	(1.10–1.70)
	COPDe	1266	50	12465	4.0	3.73	(2.41–5.79)
50–59 years	No COPD	5292	123	49787	2.5	1.00	(reference)
	COPD	2646	79	24917	3.2	1.16	(1.00–1.35)
	COPDe	1323	89	12312	7.2	2.88	(2.16–3.86)
60–69 years	No COPD	5744	263	50401	5.2	1.00	(reference)
	COPD	2872	159	26638	6.0	1.04	(0.94–1.15)
	COPDe	1436	119	12363	9.6	1.71	(1.35–2.16)
70–79 years	No COPD	2584	135	17405	7.8	1.00	(reference)
	COPD	1292	75	10307	7.3	1.03	(0.89–1.19)
	COPDe	646	50	4518	11.1	1.73	(1.23–2.42)
0 co-morbidity	No COPD	5249	99	40532	2.4	1.00	(reference)
	COPD	1284	36	10600	3.4	1.17	(0.96–1.45)
	COPDe	627	37	4744	7.8	2.68	(1.74–4.11)
1 co-morbidity	No COPD	5527	188	49963	3.8	1.00	(reference)
	COPD	2279	77	20501	3.8	1.02	(0.87–1.19)
	COPDe	1073	71	8965	7.9	2.52	(1.86–3.42)
2 co-morbidities	No COPD	4219	135	40313	3.3	1.00	(reference)
	COPD	2404	98	22626	4.3	1.08	(0.95–1.24)
	COPDe	1200	92	10570	8.7	2.19	(1.65–2.90)
≥ 3 co-morbidities	No COPD	3689	141	37065	3.8	1.00	(reference)
	COPD	3375	149	33481	4.5	1.08	(0.97–1.20)
	COPDe	1771	108	17380	6.2	1.81	(1.45–2.26)

CI = confidence interval; COPD = chronic obstructive pulmonary disease; e = exacerbations; HR = hazard ratio.

^a^Adjusted for all covariates listed in Table 1.

^b^Per 1000 person-years.

^c^Invasive treatments for patients with COPD or COPDe was included.

Compared with patients without COPD, diabetic patients with previous COPDe more frequently were male, were older, had low income status, and had higher proportions of hypertension, mental disorders, ischemic heart disease, stroke, heart failure, dementia, hyperlipidemia, anemia, Parkinson’s disease, atrial fibrillation, liver cirrhosis, peripheral vascular disease, and renal dialysis (Table 3) (all p<0.001).

**Table 3:** Characteristics of hospitalized diabetic patients with and without COPD history.

	No COPD N = 308709		COPD N = 72554		COPDe N = 14253		*P* value
Gender	n	(%)	n	(%)	N	(%)	<0.0001
Female	131332	(42.5)	31734	(43.7)	5210	(36.6)	
Male	177377	(57.5)	40820	(56.3)	9043	(63.4)	
Age, years							<0.0001
40–49	61709	(20.0)	6285	(8.7)	1002	(7.0)	
50–59	97039	(31.4)	14726	(20.3)	2168	(15.2)	
60–69	84093	(27.2)	22117	(30.5)	3796	(26.6)	
70–79	65868	(21.3)	29426	(40.6)	7287	(51.1)	
Low income	13676	(4.4)	4335	(6.0)	1211	(8.5)	<0.0001
Co-morbidities							
Hypertension	113546	(36.8)	36863	(50.8)	7087	(49.7)	<0.0001
Mental disorder	41068	(13.3)	15923	(22.0)	3402	(23.9)	<0.0001
Ischemic heart disease	31594	(10.2)	14787	(20.4)	3241	(22.7)	<0.0001
Stroke	30325	(9.8)	10939	(15.1)	2800	(19.6)	<0.0001
Heart failure	4579	(1.5)	4199	(5.8)	1569	(11.0)	<0.0001
Dementia	4020	(1.3)	2179	(3.0)	657	(4.6)	<0.0001
Hyperlipidemia	26809	(8.7)	8670	(12.0)	1146	(8.0)	<0.0001
Anemia	5203	(1.7)	2104	(2.9)	429	(3.0)	<0.0001
Parkinson's disease	2652	(0.9)	1327	(1.8)	368	(2.6)	<0.0001
Atrial fibrillation	1857	(0.6)	1107	(1.5)	321	(2.3)	<0.0001
Liver cirrhosis	7704	(2.5)	1767	(2.4)	336	(2.4)	0.4085
Peripheral vascular disease	2365	(0.8)	945	(1.3)	173	(1.2)	<0.0001
Renal dialysis	2610	(0.9)	623	(0.9)	157	(1.1)	0.0052

COPD = chronic obstructive pulmonary disease; e = exacerbations.

Concerning adverse outcomes during admissions due to diabetes (Table 4), patients with COPDe had a higher risk of pneumonia (OR 3.28, 95% CI 3.13–3.43) and admission to intensive care (OR 1.32, 95% CI 1.26–1.39). Diabetic patients with COPDe had longer length of hospital stays (16.8±66.8 vs. 10.5±48.1 days, p<0.0001) and higher medical expenditures (2838±8983 vs. 2157±5243 US dollars, p<0.0001) than those without COPD. Mortality after diabetes hospitalization was also significantly associated with history of COPDe (OR 2.06, 95% CI 1.88–2.25).

**Table 4:** Outcomes during diabetes hospitalization in patients with COPD.

	No COPD		COPD		COPDe		Risk for COPD		Risk for COPDe	
	Events	(%)	Events	(%)	Events	(%)	OR	(95% CI)^a^	OR	(95% CI)^a^
Pneumonia	19848	(6.4)	9841	(13.6)	3110	(21.8)	2.04	(1.98–2.10)	3.28	(3.13–3.43)
ICU	29527	(9.6)	7973	(11.0)	2183	(15.3)	1.01	(0.98–1.04)	1.32	(1.26–1.39)
Mortality	5114	(1.7)	1778	(2.5)	655	(4.6)	1.23	(1.16–1.30)	2.06	(1.88–2.25)
ME, US dollars^b^	2157±5243		2292±6482		2838±8983		p<0.0001		p<0.0001	
Length of stay, days^b^	10.5±48.1		11.6±48.8		16.8±66.8		p<0.0001		p<0.0001	

CI = confidence interval; COPD = chronic obstructive pulmonary disease; e = exacerbation; ME = medical expenditure; OR = odds ratio.

^a^Adjusted for all covariates listed in Table 3.

^b^Mean±SD

The adjusted ORs for COPD patients developing pneumonia after pre-admission for diabetes at 3 months, 6 months, 12 months, and 18 months were 2.77 (95% CI 2.67–2.86), 2.49 (95% CI 2.41–2.57), 2.26 (95% CI 2.20–2.33), and 2.12 (95% CI 2.06–2.18), respectively (Table 5). The risks of mortality and ICU stay associated with COPD also decreased with the time course of COPDe occurrence. Similar results regarding risk of post-diabetes pneumonia, mortality and ICU stay were also found in patients with COPDe. The risk of post-diabetes pneumonia was associated with the occurrences of COPDe within pre-admission for diabetes at 3 months (OR 3.78, 95% CI 3.56–4.02), 6 months (OR 3.46, 95% CI 3.28–3.66), 12 months (OR 3.37, 95% CI 3.21–3.54), and 18 months (OR 3.29, 95% CI 3.13–3.44). The ORs of post-diabetes pneumonia, mortality and ICU stay associated with respiratory invasive treatment in patients with COPDe were 3.30 (95% CI 3.10–3.53), 0.92 (95% CI 0.77–1.10), and 1.52 (95% CI 1.41–1.63), respectively.

**Table 5:** Time effects of COPD on the outcomes of diabetes admission.

		Pneumonia		Mortality		ICU stay	
History of COPD	n	OR	(95% CI)	OR	(95% CI)	OR	(95% CI)
No COPD	308709	1.00	(reference)	1.00	(reference)	1.00	(reference)
COPD occurred							
Pre-admission 3 month	29390	2.77	(2.67–2.86)	1.41	(1.31–1.52)	1.09	(1.05–1.13)
Pre-admission 6 month	38499	2.49	(2.41–2.57)	1.38	(1.28–1.48)	1.06	(1.02–1.09)
Pre-admission 12 month	52461	2.26	(2.20–2.33)	1.31	(1.23–1.40)	1.04	(1.00–1.07)
Pre-admission 18 month	63310	2.12	(2.06–2.18)	1.27	(1.20–1.35)	1.02	(0.99–1.05)
COPDe occurred							
Pre-admission 3 month	6520	3.78	(3.56–4.02)	2.26	(2.01–2.55)	1.45	(1.36–1.56)
Pre-admission 6 month	8350	3.46	(3.28–3.66)	2.27	(2.04–2.52)	1.43	(1.35–1.52)
Pre-admission 12 month	10858	3.37	(3.21–3.54)	2.21	(2.01–2.43)	1.39	(1.31–1.47)
Pre-admission 18 month	12733	3.29	(3.13–3.44)	2.10	(1.92–2.31)	1.36	(1.29–1.43)
With invasive treatment	5896	3.30	(3.10–3.53)	0.92	(0.77–1.10)	1.52	(1.41–1.63)

CI = confidence interval; COPD = chronic obstructive pulmonary disease; e = exacerbation; ICU = intensive care unit; OR = odds ratio.

## Discussion

Our retrospective cohort study showed that COPD patients with and without exacerbations showed significantly increased risk of developing diabetes compared with those without COPD. The nested cohort study showed diabetic patients with history of COPD were significantly associated with increased pneumonia, admission to intensive care, prolonged length of stay, increased medical expenditure and mortality. The results of our studies were consistent with previous reports. [11–16]

Exacerbation is critically important in the natural history and clinical outcome for COPD patients. Our study showed that COPD patients with or without exacerbations were associated with higher risk of developing diabetes, and COPD *per se* impacts diabetes outcomes significantly. Patients experiencing frequent exacerbations were at higher risk for declined lung function and increased mortality [22–24]. Previous report also suggested that most COPD exacerbations are due to lower respiratory tract infections [25], which significantly worsened outcomes in COPD patients in terms of increased exacerbation rate and mortality [26].

Comorbidities including hypertension, hyperlipidemia, stroke and cardiovascular disease were known as independent factors associated with diabetes also commonly coexisting in patients with COPD [5–7,11,13,27].To reduce confounding effects, we used multivariate regression models to adjust comorbid conditions and calculated the risk of diabetes in patients with COPD. Age, gender and socioeconomic status were also considered as potential confounding factors associated with COPD and diabetes [12]. All these characteristics were adjusted in the multivariate regression models. Although previous cohort studies used nationwide data to analyze the risk of diabetes in COPD patients, they were limited by inadequate adjustment for potential confounders [15,16]. The present study showed that COPD with exacerbations was associated with risk of developing diabetes in various age groups, co-morbidities and both sexes. However, the association was weaker in subjects with more than three co-morbidities. It is possible that more numerous and complex co-morbidities may dilute the impact of COPD on the risk of diabetes. Compared with non-COPD group, COPDe patients with invasive respiratory treatment did not have increased diabetes risk and post-diabetes mortality in this study. This non-significant association may be due to the beneficial effects of invasive respiratory treatment for patients with COPDe. However, the phenomenon needs future clinical trials for proving the beneficial effects of invasive respiratory treatment.

Although the mechanism for increased risk of diabetes in COPD remains unclear, we suggest that systemic inflammation is a plausible explanation. In patients with COPD, there is much evidence that the serum levels of inflammatory mediators are increased, including tumor necrosis factor alpha (TNF-α), interleukin-6 (IL-6) or C reactive protein (CRP) [28,29]. High levels of TNF-α may interfere with glucose metabolism and insulin sensitivity and increase the risk of new onset diabetes [30, 31]. Elevated levels of IL-6 and CRP have been shown to predict the development of type 2 diabetes [28, 29].

Another possible explanation for increased risk of diabetes in COPD patients might relate to COPD medications. Current international guidelines suggest systemic glucocorticoid therapy, at least a 5-day course, to manage COPD exacerbations [32]. Yet prolonged exposure to corticosteroids is known to lead to substantial side effects in COPD patients, even death [33]. In addition, steroid therapy for COPD can lead to the development or worsening of diabetes [34,35], although controversy surrounds this observation [36,37]. Whether high-dose and/or long-term steroid use in COPD patients causes type 2 diabetes needs further investigation. Oxidative stress is considered an imbalance between oxidants and antioxidants. In COPD patients, either when stable or during exacerbations, oxidative stress was induced mainly by inhaled oxidants such cigarette smoke or pollution [38]. Oxidative stress, mainly smoke-induced in COPD patients, could cause insulin resistance in type 2 diabetes [39].

A previous study found that diabetes is associated with an increased risk of pulmonary infections, disease exacerbations and worsened COPD outcomes [31]. On the other hand, we found that COPD may be considered a novel risk factor for new onset diabetes and this phenomenon may via multiple mechanisms, including steroids therapy and oxidative stress [28,29]. The further investigation is needed to clarify the link between COPD and diabetes.

This study has some limitations. First, we used insurance claims data that lacked detailed information on sociodemographic and lifestyle factors, hormonal status and biomedical measures [6,7]. Second, pulmonary function data were not available, so the severity of COPD was not classified using Global Initiative for Obstructive Lung Disease criteria [40]. When interpreting the findings of this study should be cautioned because the unavailable data of lung function test is a very important limitation. However, exacerbations are generally considered to become more frequent as the severity of underlying COPD increases [41] and findings from this study cannot be compared to those using Global Initiative for Obstructive Lung Disease criteria for disease staging [12,40]. Third, although we used multivariate adjustment to control for confounders, residual confounding is always possible.

In conclusion, COPD is associated with higher risks of developing diabetes or post-diabetes pneumonia, mortality. However, the real mechanism between COPD and diabetes needs further basic lab data and clinical investigations.

## Abbreviations

CI: confidence interval
COPD: chronic obstructive pulmonary disease
HR: hazard ratio
*ICD-9-CM*: *International Classification of Diseases*, *9th Revision*, *Clinical Modification*
OR: odds ratio
