# Correction: Diabetes risks and outcomes in chronic obstructive pulmonary disease patients: Two nationwide population-based retrospective cohort studies

**DOI:** 10.1371/journal.pone.0190289

**Published:** 2017-12-20

**Authors:** The PLOS ONE Staff

There is an error in affiliation 1 for authors Chao-Shun Lin, Chih-Chung Liu, Ta-Liang Chen, and Chien-Chang Liao. Affiliation 1 should be: Department of Anesthesiology, School of Medicine, College of Medicine, Taipei Medical University, Taipei, Taiwan.

There is an error in affiliation 3 for authors Chao-Shun Lin, Chih-Chung Liu, Ta-Liang Chen, and Chien-Chang Liao. Affiliation 3 should be: Department of Anesthesiology, Taipei Medical University Hospital, Taipei, Taiwan. The publisher apologizes for the errors.
